# Methoprene and Temperature Effects on Caste Differentiation and Protein Composition in the Formosan Subterranean Termite, *Coptotermes formosanus*

**DOI:** 10.1673/031.012.1801

**Published:** 2012-02-06

**Authors:** Matthew R. Tarver, Christopher B. Florane, Dunhua Zhang, Casey Grimm, Alan R. Lax

**Affiliations:** ^1^ Formosan Subterranean Termite Research Unit, USDA-ARS-SRRC, New Orleans LA; ^2^ Food Processing and Sensory Quality Research Unit, USDA-ARS-SRRC, New Orleans LA

**Keywords:** hexamerin, LC/MS/MS, methoprene, phenotypic bioassay, social insect

## Abstract

The utilization of multiple castes is a shared feature of social insects. In termites, multiple extrinsic factors have been shown to impact caste differentiation; for example, increased temperature has been shown to increase soldier production. Also, application of exogenous methoprene has also been demonstrated to increase soldier production. The objective of this investigation was to examine and correlate the effects of temperature variation and methoprene treatments on termite caste differentiation, and identify the resulting changes in protein levels. Our results indicate that worker—to—soldier differentiation is modulated by temperature, where a greater number of soldiers developed at a higher rate at higher temperatures compared to lower temperatures. We analyzed total protein by sodium dodecyl sulfate Polyacrylamide gel electrophoresis and N-terminal sequencing and found several changes. Specifically, four proteins affected by temperature change were identified: Hexamerin-1, Hexamerin-2, Endo-beta 1,4 glucanase, and myosin. These proteins were further examined for their response to temperature, assay length (time), and exposure to the juvenile hormone analog methoprene. Hexamerin-1 protein showed a temperature—and assay length—dependent effect, while Hexamerin-2, Endo-beta 1, 4 glucanase, and myosin protein levels were all affected by temperature, assay length, and exposure to methoprene. Our analysis allows the correlation of temperature, assay length, and presence of methoprene with specific changes in protein levels that occur during caste differentiation. These results can be directly applied to better understand the complex developmental factors that control termite differentiation and guide the use of juvenile hormone analogs to maximize efficiency of termite eradication in the field.

## Introduction

Termites are significant and costly pests throughout the world; each year it is estimated that termites are responsible for billions of dollars in damage and control costs (Su 2002). One unique feature contributing to the success of these household pests is that they are one of the few organisms to have evolved eusocial behavior. Termite colonies are made up of tens of thousand of individuals, all working together. All social insects share three main characteristics: overlap of generations, cooperative care of young, and a caste system (Wilson 1971). Eusocial insects use multiple castes to perform different tasks within a colony. This task partitioning results in an increase in colony efficiency (Waibel et al. 2006).

Termite colonies are made up of three main behavioral/phenotypic castes: workers, soldiers, and reproductives (Wilson 1971). Other minor castes can be present (i.e., second and third form reproductives, presoldiers, alates). The differentiation of termites from one caste to another is dependent on a number of intrinsic and extrinsic factors (Lenz 1976; Henderson 1998; Mao et al. 2005; Scharf et al. 2007). Extrinsic factors may include ambient temperature, moisture, primer pheromones, etc. (Henderson 1998; Cabrera and Kamble 2001; Park and Raina 2004, 2005; Mao et al. 2005; Liu et al. 2005; Scharf et al. 2007; Dong et al. 2009).

Temperature has been shown to play a major role in the development and caste differentiation of termites (Fei and Henderson 2002; Liu et al. 2005; Scharf et al. 2007). Fei and Henderson (2002) investigated the effect of temperature on the formation of soldiers in *Coptotermes formosanus* Shiraki (Isoptera: Rhinotermitidae) in groups starting with 100% workers. Their results show that soldier development was temperature—dependent, with a greater number of soldiers differentiating at higher temperatures. Liu et al. (2005) investigated the effects of both temperature and food quality on juvenile hormone (JH) titer and soldier formation. Workers fed on a high quality diet had a greater percentage of soldier differentiation and higher JH titers. They also showed that JH titer increased with temperature, showing that at higher temperatures there was a greater level of JH and a greater number of presoldiers formed. In termites, JH titer correlates with presoldier production, and higher levels of JH in workers leads to the formation of soldiers (Park and Raina 2004; Mao et al. 2004). Research done by Scharf et al. (2007) looked at the two major hemolymph proteins Hexamerin 1 (Hex-1) and Hexamerin 2 (Hex-2) in response to temperature and food consumption. They showed that temperature and feeding were correlated with Hex-1/Hex-2 protein levels and associated with presoldier/soldier development in *Reticulitermes flavipes.* The higher the temperature, the greater number of presoldiers/soldiers that were formed through Hex1/Hex2 proteins modulating JH binding (Zhou et al. 2006, 2007; Scharf et al. 2007) The number of soldiers and alates within a colony has also been shown to be temperature—dependent, with more soldiers and alates in the spring and summer months (Haverty 1981; Waller and La Fage 1988; Liu et al 2005).

The present research further investigates the influence of temperature on *C. formosanus* presoldier formation and protein abundance, and attempts to identify effects that can be correlated to temperature and time. This study aims (1) to examine the extrinsic factor— temperature—on termite caste differentiation and protein abundance, (2) to correlate total protein abundance across temperature, assay length (time), and JH analog treatment, and (3) to identify *C. formosanus* genes/proteins that respond to changes in temperature, JH analog, and time. The findings presented here provide evidence that *C. formosanus* soldier differentiation is potentially controlled by temperature and multiple proteins temporally are affected by temperature and treatment with a JH analog.

## Materials and Methods

### Termites

*Coptotermes formosanus* termite colony fractions were collected from multiple locations within City Park, New Orleans, Louisiana, USA. In—ground traps consisted of irrigation boxes filled with spruce (*Picea* sp.) wood and cardboard rolls. In—ground traps were placed in 1999 and have since been regularly monitored. Collections were brought back to the laboratory, separated from the collection material, placed in plastic containers with moist spruce, and kept in an incubator at 25 °C. Colonies had been kept in the laboratory for at least two weeks, but not longer than three months before use in experiments. Five different colonies were used in these experiments. Colonies were identified as *C. formosanus* by 16s mitochondrial rDNA sequencing (Szalanski et al. 2003).

### Phenotypic bioassays

Presoldier phenotypic bioassays were performed similarly to Scharf et al. (2003b) and Tarver et al. (2009), but with slight modification. Filter paper (42.5 mm) was treated either with methoprene (16 µg in acetone) (Sigma-Aldrich PS1040, www.sigmaaldrich.com) or acetone. A concentration of 16 µg per assay replication of methoprene was determined by preliminary concentration responses (MRT and CF, unpublished data). Filter paper was allowed to air dry and then placed into plastic Petri dishes (50 mm × 9 mm). Filter paper was then moistened with 180 µl of de—ionized H_2_O. Twenty worker termites were placed into each dish. Termites were carefully chosen to use only the worker caste (greater than third instar). Dishes were then placed into secondary containers containing moist paper towels to maintain humidity. Separate containers were then placed into five different incubators, each set at a different temperature (20, 23, 25, 28, 31 °C). Temperatures were chosen based on temperatures termites might encounter in the field. Temperature was also monitored by using a thermometer and checked regularly. Mortality and caste differentiation was monitored every five days for a total of 25 days. Water was added as needed. Experiments were replicated across five colonies; colony 1 had ten replicates per treatment, while colonies 2–5 had five replicates per treatment. Additional replicates for each treatment were added to colony 2 for protein analysis (three reps per day per treatment).

### Protein analysis

The additional replicates from colony 2 were destructively sampled at multiple time points (0, 1, 5, 10, 15 days) for protein analysis. All worker termites from each treatment were collected and placed into 1.5 mL Eppendorf tubes and immediately frozen at -80 °C. Once all of the samples were collected from each time point, each sample was ground in 600 µL of 1% phosphate buffered saline (Sigma-Aldrich, P-4417) and centrifuged at 14,000 g for 10 min. The supernatant fraction was collected and saved. Total protein levels were estimated using a Quick Start Bradford Protein Assay Kit (Bio-Rad Laboratories, www.bio-rad.com) using bovine serum albumin standards included in the kit.

An aliquot of 10 µg of total protein was loaded into each well of SDS-PAGE gels (Life Technologies, www.invitrogen.com) with 4–12 % gradient and run using an Invitrogen X-cell SureLock Mini-Cell system (Life Technologies). Each gel was run with in—gel bovine serum albumin standards at concentrations ranging from 0.125 to 0.50 (Scharf et al. 2007). The standards were included to allow in—gel comparisons using densitometry analysis of target proteins. Gels were stained using Invitrogen SimplyBlue SafeStain according to manufacturer protocol (Life Technologies). After staining, gels were scanned and protein bands of interest were compared to the in—gel standards for abundance (TotalLab Limited, www.totallab.com). For N-terminal sequencing analysis, SDS-PAGE gels were electroblotted to PVDF membrane (0.45 µm, Invitrogen) and stained with 0.1 % Ponceau S (in 5 % acetic acid). Bands of interest were excised for N-terminal sequencing (Baylor College of Medicine, www.bcm.edu).

### Peptide analysis

Abundant protein bands were excised and digested using the In-Gel Tryptic Digestion Kit (Thermo Scientific, www.thermoscientific.com, No. 89871). The tryptic digests were analyzed via LC/MS/MS, using an Agilent 1260 LC system, an Agilent Chip-cube interface and an Agilent 6520 quadrupole time—of—flight tandem mass spectrometer (Agilent Technologies, www.aligent.com). Chromatographic separation was accomplished using a Chip consisting of a 40 nL enrichment column and a 43 mm analytical column packed with C18, 5 µm beads with 300A pores. One µL aliquots of the sample were transferred to the enrichment column via the 1260 capillary pump operating at a flow of 4 µL/min. The 1260 nano pump was operated at a flow rate of 600 nL/min. An initial gradient (Solvent A - 100% H_2_O, 0.1% formic acid; Solvent B 90% acetonitrile (ACN), 10% H_2_O, and 0.1% formic acid) of 97% Solvent A was changed to 30% Solvent A at 12 min, 0% at 13 min, 100% at 14 min, and 0% at 15 min. A post run time of 4 min was employed for column equilibration.

The mass spectrometry source was operated at 300 °C with 5 L/min N_2_ flow and a fragmentor voltage of 175 V. N_2_ was used as the collision gas with collision energy varied as a function of mass and charge using a slope of 3.7 V/100 Da and an offset of 2.5 V. Both quadrupole and TOF were operated in positive ion mode. Reference compounds of 322.048121 Da and 1221.990637 Da were continually leaked into the source for mass calibration. An initial mass spectrometry scan was performed from m/z 300 to 1600 and up to three multiply charged ions were selected for MS/MS analysis. Following the initial run, a second injection was made excluding ions previously targeted for MS/MS analysis.

Data files were transferred to an Agilent workstation equipped with Spectrum Mill software (Agilent Technologies). The raw MS/MS data files were extracted, sequenced, and searched against the TrEMBL database. Data was obtained from the TrEMBl Database Release 2010_11. Summed MS/MS search score is based upon a perfect score of 25 for an individual peptide, with values > 13 generally considered to be excellent fits.

### Statistical analyses

Data from the phenotypic bioassays were analyzed using an ANOVA for the main effects of percent cumulative presoldier formation at day 25, colony, temperature, and treatment (JMP, www.jmp.com). Additional analyses of pairwise *t*-tests between methoprene and non—methoprene treatments were also performed. This method was repeated for percent mortality but the addition of the interaction of temperature*treatment was added to the effects test.

Data from protein densitometry was analyzed by ANOVA testing for the main effects of temperature, treatment, day, and the interactions between the effects (Table 1).

## Results

### Temperature dependent bioassays

**Figure 1.**
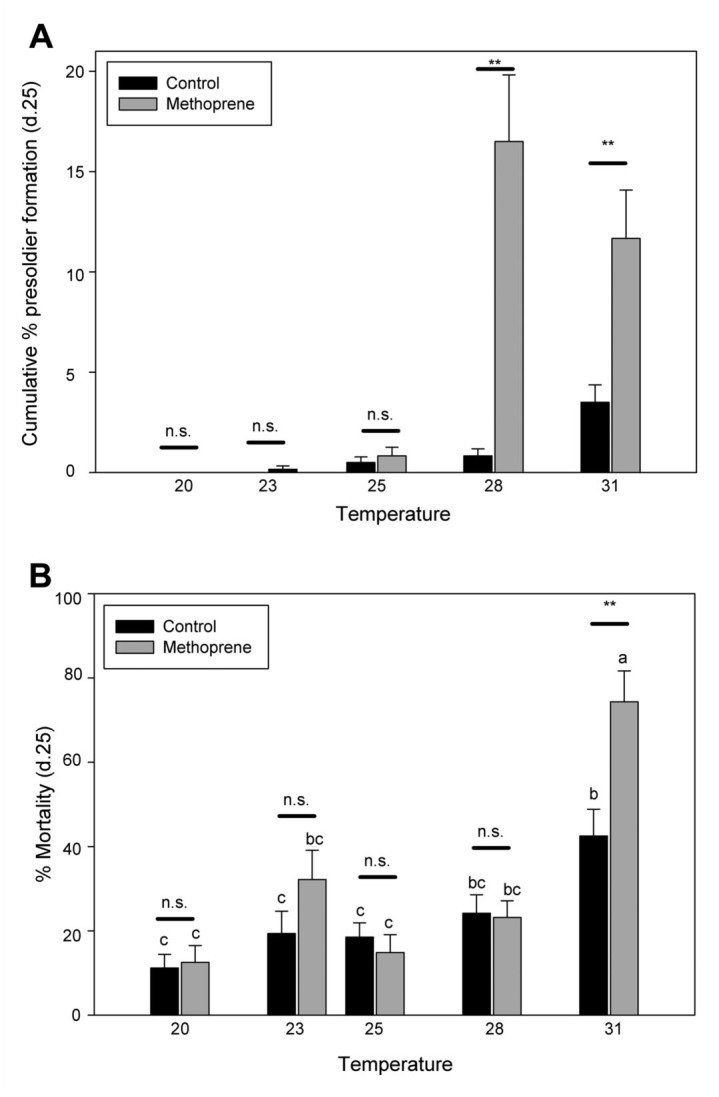
Impact of multiple temperatures on caste differentiation and mortality. Results of multiple temperatures 20–31 °C on (A) cumulative presoldier formation and (B) mortality. Asterisks (*) indicate a significant difference (*t*-test) with and without the JH-analog, methoprene. Different letters represent a significant difference (*p* < 0.05). N.S. indicates there was no difference between the two treatments. High quality figures are available online.

*Coptotermes formosanus* soldier induction was monitored across multiple temperatures (20, 23, 25, 28, 31 °C) in the presence and absence of the juvenile hormone analog, methoprene. Results across five different colonies indicate an increase in presoldier formation with an increase in temperature (ANOVA whole model - df = 9,299, *F* = 18.7213, *p* < 0.01; temperature - df = 4, *F* = 20.8044, *p* < 0.01; treatment - df = 1, *F* = 31.814, *p* < 0.01; colony - df = 4, *F* = 13.3582, *p* < 0.01) (Figure 1a). As expected, presoldier formation also increased with methoprene treatment (non—methoprene average 1.00 ± 0.15, methoprene 5.00 ± 0.07). The addition of methoprene significantly increased presoldier formation across temperatures. There was a significant difference in presoldier formation with methoprene treatment at 28 and 31 °C compared to the non—methoprene treatment (*p* < 0.05) (Figure la). Presoldier differentiation was highest in the 28 °C methoprene treatment groups (16.5 ± 3.32). There was also an increase in presoldier differentiation in the non—methoprene treatments, with the greatest presoldier formation at the highest temperature (31 °C) (3.5 ± 0.87).

**Table 1.**
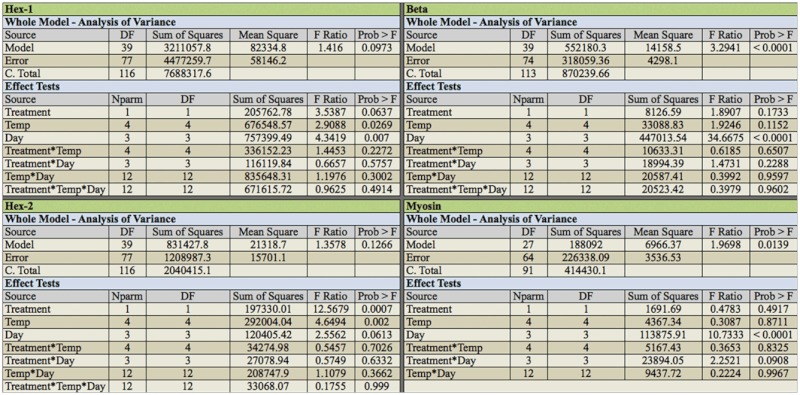
Whole model and effect tests for estimated protein abundance.

The overall mortality across all treatments and temperatures was (27.27 ± 1.93 SEM). The increase in temperature also raised the mortality rate as did the addition of methoprene in the higher temperatures (ANOVA whole model - df = 13,299, *F* = 10.5408, *p* < 0.01; temperature - df = 4, *F* = 25.3381, *p* < 0.01; treatment - df = 1, *F* = 6.4797, *p* < 0.01; colony - df = 4, *F* = 3.2575, p < 0.01; treatment*temperature - df = 4, *F* = 4.0421, *p* < 0.01) (Figure 1b). As a result the increase in mortality reduced the number of presoldiers formed in the higher temperature treatments.

### Temperature and methoprene effect on protein abundance

**Figure 2.**
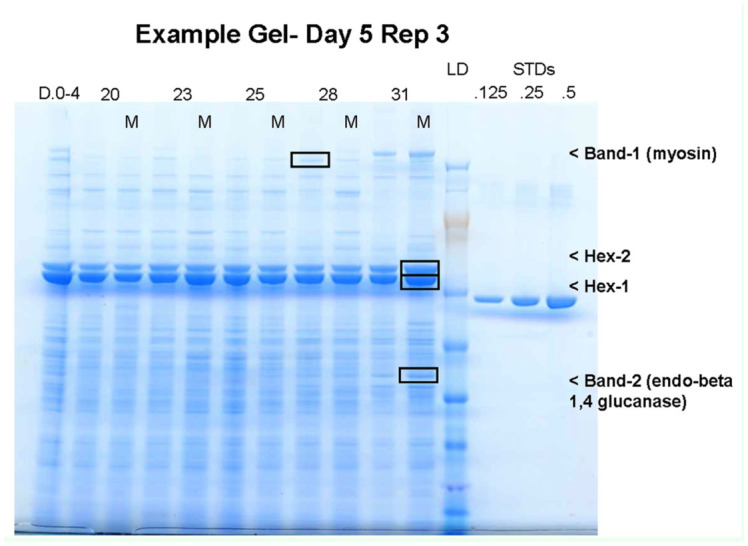
Representative SDS-PAGE gel. A typical gel image from the protein abundance experiment (Day 5, replicate-3). Each temperature with and without methoprene is shown. Molecular weight marker and three in-gel bovine serum albumin standards (0.125, 0.25, 0.5) were included for estimation of relative protein abundance. Hexamerin 1, 2, Band #1, and Band #2 are indicated. High quality figures are available online.

Side by side samples were destructively sampled at multiple time points (days 1, 5, and 10) and total extractable protein was analyzed by SDS-PAGE (Figure 2). Multiple protein bands were visualized and the most abundant proteins showing differing amounts with treatments or times were chosen for peptide sequencing. Four different protein bands were focused on for this research. Each of the four bands was measured for differences in abundance using densitometry (Figure 2). N-terminal sequencing of two prominent protein bands revealed that one had the N-terminal amino acid sequence YPGTSPSQ and the other had DHHIEKKMAD. The first one matched one of our EST cDNA sequence, coding for Hexamarin-2 (#1424505), and the second closely matched the first 10 amino acids of *R. flavipes* mature Hexamarin-1 (AAU20851). The apparent molecular weights of these two proteins in SDS-PAGE (Figure 2) were about 85 kDa (Hex-2) and 80 kDa (Hex-1), respectively, which are similar to those of *R. flavipes.* Two other bands were excised and identified using tryptic digest followed by LC/MS/MS. Unknown protein band #1 (216 KD) was putatively identified as myosin (%12 coverage, MS/MS score - 379.03, accession number B4LUG2 ) (Table 2). Unknown protein band #2 was identified as endo-beta 1,4 glucanase (Beta 1,4, 50 kDa, 15% coverage, MS/MS score - 67.88, accession number Q9BH22)(Table 2).

**Table 2.**
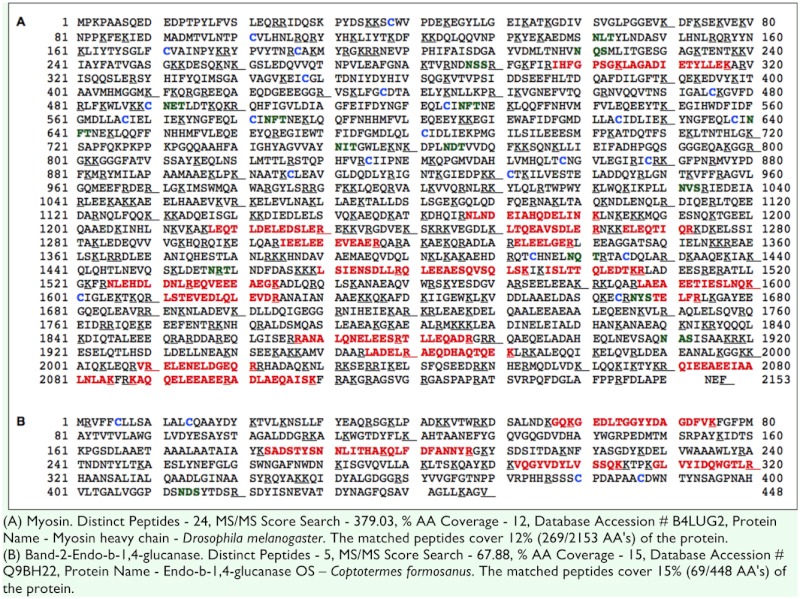
Number of distinct peptides, MS/MS score, and sequence coverage on matching peptides from *Coptotermes formosanus* Bands 1 and 2.

**Figure 3.**
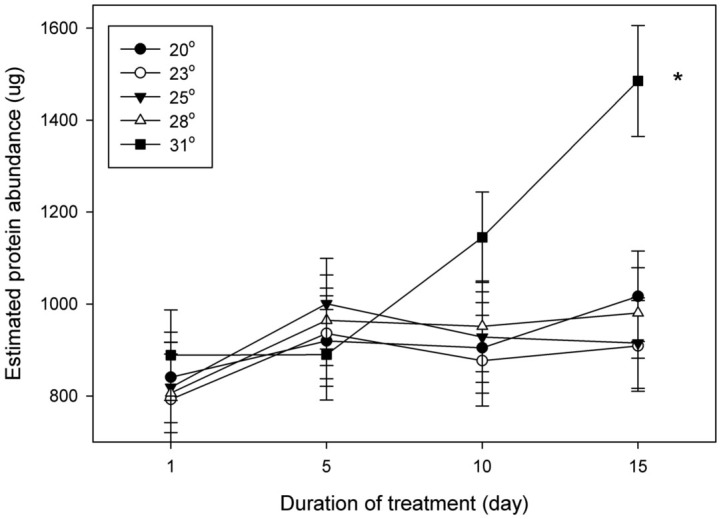
Hexamerin I protein abundance in relationship to temperature and day. Estimated Hexamerin I protein abundance of each temperature across days. Termites subjected to 15 days at 31 °C had significantly higher protein abundance than other temperatures (*p* < 0.05). High quality figures are available online.

Using densitometry analysis relative to in—gel standards, Hex-1, Hex-2, Beta, and Myosin abundance were measured. Hex-1 protein abundance was affected by temperature and day, with an increase in protein abundance at the later days and greater temperature (Table 1). When the temperature and day effects were separated, it was shown that Hex-1 protein levels only increased in the 31 °C treatment at day 15 (Figure 3). These results indicate the Hex-1 protein increases at later time points and at higher temperature, the same treatments that resulted in greater presoldier formation.

**Figure 4.**
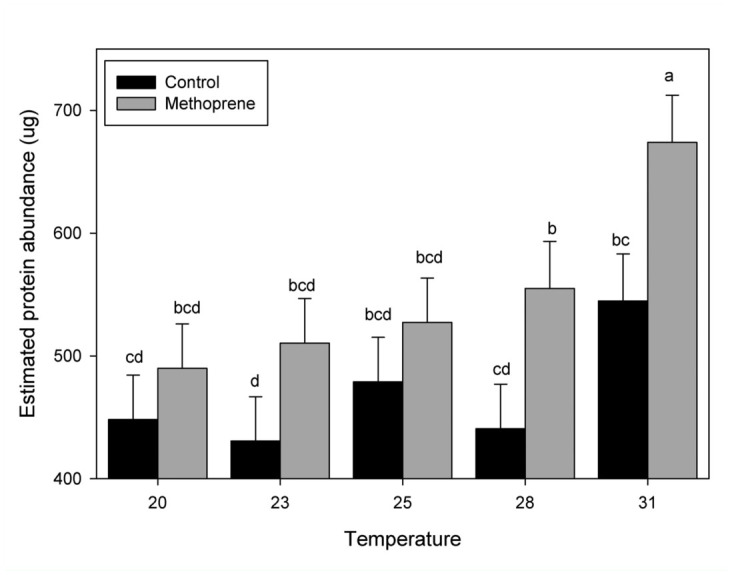
Hexamerin 2 protein abundance in relationship to temperature and treatment. Estimated Hexamerin 2 protein abundance for each temperature tested with and with out methoprene. Bars with different letters represent a significant difference (*p* < 0.05). High quality figures are available online.

Hexamerin-2 protein was shown to be affected by treatment (non—methoprene or methoprene) and temperature (Table 1). Hex2 abundance levels increased with methoprene treatment and at the highest temperature (31 °C). When the temperature and treatment effects were separated Hex-2 levels increased in the methoprene treatments and increased only at the highest temperature (Figure 4).

**Figure 5.**
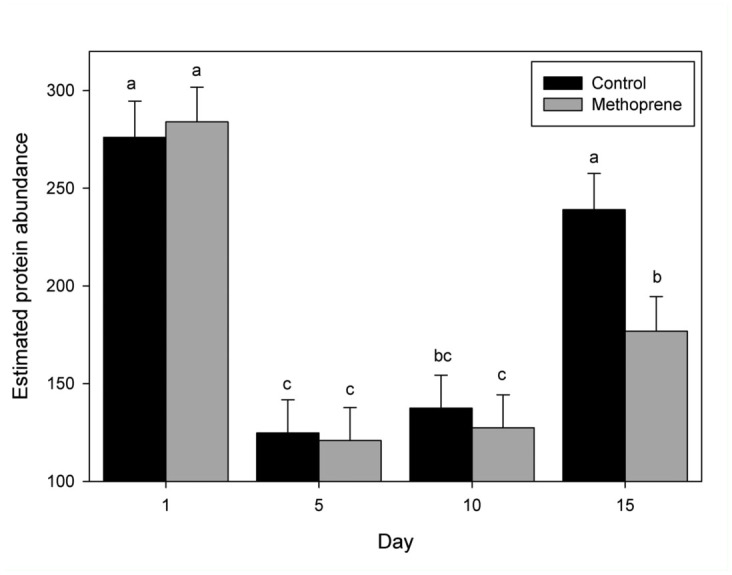
Band-2 (endo-beta 1,4 glucanase) protein abundance in relationship to day and treatment. Estimated Band-2 (endo-beta 1,4 glucanase) protein abundance by day with and without methoprene. Band-2 LC/MS/MS data was best fit to endo-beta 1,4, glucanase from *Coptotermes formosanus.* Bars with different letters represent a significant difference (*p* < 0.05). High quality figures are available online.

**Figure 6.**
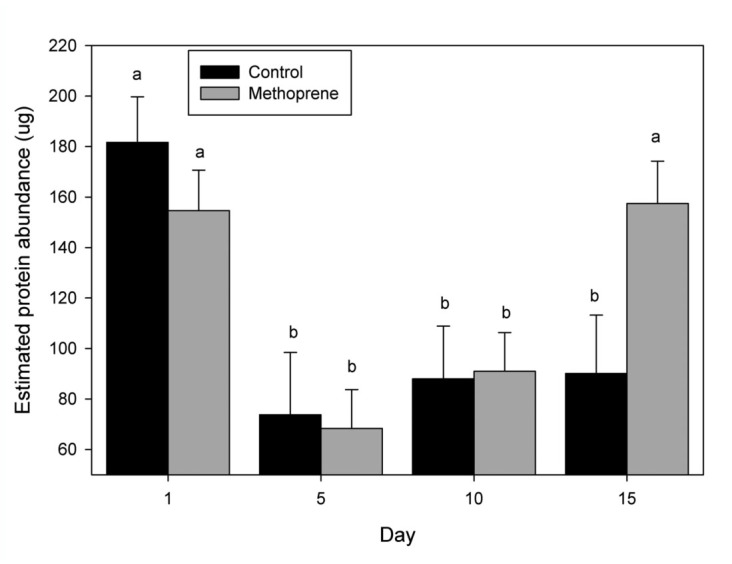
Band-1 (myosin) protein abundance in relationship to day and treatment. Estimated Band-1 (myosin) protein abundance by day with and without methoprene. Band-1 LC/MS/MS data was best fit to myosin from *Drosophila melanogaster.* Bars with different letters represent a significant difference (*p* < 0.05). High quality figures are available online.

Endo-beta-1,4 glucanase and myosin protein levels were affected by day (Table 1). When the day and treatment effects were separated Endo-beta-1,4 glucanase (beta-1,4) protein levels were highest at day 1 and were reduced through days 5 and 10, and increased significantly in day 15 non—methoprene treatments (Figure 5). When the day and treatment effects were separated for myosin protein levels, as with beta-1,4 they were the highest at day 1 and were reduced through days 5 and 10. But unlike beta-1,4, there was a significant increase in myosin protein levels at day 15 in the presence of methoprene treatments (Figure 6).

## Discussion

Termite caste differentiation is a complex process and the details of this biological process are still largely unknown. Because termites are cryptic and their colonies secluded, it makes it more difficult to observe their unique life style compared to other social insects (i.e., ants, bees, and wasps). Termite caste differentiation has been shown to be modulated by a multitude of extrinsic and intrinsic factors (Howard and Haverty 1981; Waller and La Fage 1988; Horiuchi et al. 2002; Fei and Henderson 2002; Liu et al. 2005; Park and Raina 2004; Mao et al. 2005; Scharf et al. 2007; Tarver et al. 2009, 2010).

The results presented here support previous findings that show that *C. formosanus* soldier production is modulated by temperature with a direct increase in presoldier formation as temperature increases. Presoldier percentage was found to be the greatest at 28 °C, and although there was an increase in presoldier formation at the highest temperature (31 °C), higher mortality may have had an impact at this higher temperature. No presoldiers formed at the lowest temperature (20 °C), while there were a few presoldiers in the methoprene treatment at 23 °C. These results suggest that soldier formation in colonies is less likely to occur at lower temperatures. This correlates with previous research that found an increase in soldier caste number in the spring season, suggesting a seasonal effect in caste regulation (Fei and Henderson 2002). These results also suggest that temperature factors should be considered when investigating other mediators of termite soldier formation.

The modulation of temperature has been shown to have an influence on caste differentiation and hexamerin protein levels in a related termite, *R. flavipes* (Scharf et al. 2007). Similar to the results shown here, an increase in temperature increased *R. flavipes* presoldier formation. Differences between the previous research (Scharf et al. 2007) and the research presented here are that additional temperatures were tested (five compared to two) and the Hex-1 and Hex-2 proteins were analyzed separately compared to being combined in the previous study.

By analyzing the Hex-2 and Hex-1 proteins separately we were able to determine that the Hex-2 is the protein most influenced by the JH analogue treatment, methoprene. Hex-2 only showed a significant temperature—dependent effect in the methoprene treatments, although there was a slight increase in the non—methoprene Hex-2 levels in relationship to temperature (Figure 3a). These results have a similar pattern to the presoldier formation bioassays, in which presoldier formation increased with temperature and methoprene. Hexamerin proteins have been suggested to directly bind/modulate JH levels in *R. flavipes*, thereby regulating caste differentiation (Zhou et al. 2006b, 2007; Scharf et al. 2007). Liu et al. (2005) measured JH levels in *C. formosanus* and found an increase in JHIII levels/presoldier formation with an increase in temperature.

Hexamerin-1 protein was shown to be affected by day and temperature, but there was an increase in protein abundance as assay day and temperature increased. Hex-1 in *R. flavipes* showed a unique hydrophobic tail, a putative JH binding site, and was shown to be potentially hemolymph—soluble (Zhou et al. 2006b). If *C. formosanus* Hex-1 has a similar function, perhaps the lack of fluctuation with JH analog treatment could be due to an already abundant protein in the hemolymph or the use of JH analog instead of JHIII. Further experiments determining the potential binding of *C. formosanus* Hex-1 with JH or JH analog are needed.

The protein band that had homology to endobeta-1,4 glucanase had an increase in abundance at day one with a decrease with time, but had an increase in abundance in the non—methoprene (control) treatment at day 15. This discrepancy between protein abundance at day 15 could be because a larger number of individuals were molting to soldiers in the methoprene treatments. Endoglucanases are digestive enzymes found in the termite digestive tract specifically, the endo-beta-1,4-glucanases are responsible for the cleavage of the internal beta—linked glycosidic bonds in glucose polymers (Zhou et al. 2010). This, coupled with the fact that termites molting between castes expel all contents from the gut before molting (Cleveland 1925; Raina et al. 2008), could explain the reduction in endobeta-1,4 protein at the later time point. Additional research needs to be conducted to shed light on this hypothesis.

The final protein band that was analyzed had homology to myosin, a protein component of muscle. Myosin levels were high at the early time point and then reduced through days 5 and 10. At day 15 there was an increase in protein abundance in the methoprene treatments only. This increase correlates with the increase in presoldiers. Perhaps the increase in myosin protein abundance is in response/relationship to the increase in muscle needed for the soldier caste. Interestingly, previous research looking at gene expression in *R. flavipes* also showed an increase in myosin gene expression in relationship to worker—to—soldier differentiation (Scharf et al. 2003; Tarver et al. 2010). Scharf et al. (2003) saw an increase in myosin soldier gene expression compared to worker gene expression. Results from Tarver et al. (2010) showed a significant increase in myosin gene expression in worker termites during the worker—to—soldier caste differentiation process. Together, these findings suggest that myosin plays an important role in termite soldier development. Future work combining gene expression, protein abundance, and soldier development in the same experiment needs to be carried out to functionally characterize this potentially important downstream gene.

Our gels demonstrate that other proteins are modulated by temperature and methoprene, but for these experiments we focused on Hex2 and Hex-1 based on previous knowledge, and endo-beta-1,4 and myosin because of strong visible protein abundance changes and LC/MS/MS hits. Further research to identify other differently expressed proteins is currently ongoing.

One—dimensional SDS-PAGE separates proteins based on the mass/charge ratio. The drawback of using only 1D separation is that multiple proteins might have similar ratios; therefore, there might be multiple proteins within a single band. Absolute identification of the band #1 (endo-beta-1,4) and band #2 (myosin) may not be totally conclusive because of this reason. But relative size and strong mass spectral database matches gives us confidence of their identity. Further 2D and total proteomics analysis need to be conducted to verify their identity and to identify additional proteins responsible for pre—soldier formation.

These results together, along with past research (Scharf et al. 2007) in *C. formosanus* and *R. flavipes*, suggest that temperature plays an important role in caste differentiation, and that the Hex-2 protein could have the same role as in *R. flavipes* in JH modulation, which thereby has a direct impact on worker—to—soldier caste differentiation. Finally, the changes in abundance of the putative myosin protein correlate with worker—to—presoldier transformation and strongly suggest a need for the muscle protein in the soldier termite. Discovery of proteins responsible for or associated with caste differentiation will increase our understanding of termite differentiation and could provide new targets for development of termite specific control treatments.

## Abbreviations

**LC/MS/MS**,: liquid chromatography/mass spectrometry/mass spectrometry

## References

[bibr01] Cabrera B, Kamble S (2001). Effects of decreasing thermophotoperiod on the eastern subterranean termite (Isoptera: Rhinotermitidae).. *Environmental Entomology*.

[bibr02] Cleveland LR (1925). The feeding habit of termite castes and its relation to their intestinal flagellates.. *Biological Bulletin*.

[bibr03] Dong S-L, Mao L, Henderson G (2009). Physical contact between soldier and worker is essential in soldier self—regulation of *Coptotermes formosanus* (Isoptera, Rhinotermitidae).. *Insectes Sociaux*.

[bibr04] Fei H, Henderson GR (2002). Formosan subterranean termite wood consumption and worker survival as affected by temperature and soldier proportion.. *Environmental Entomology*.

[bibr05] Haverty MI, Howard RW (1981). Production of soldiers and maintenance of soldier proportions by laboratory experimental groups of *Reticulitermes flavipes* (Kollar) and *Reticulitermes virginicus* (Banks) (Isoptera: Rhinotermitidae).. *Insect Sociaux*.

[bibr06] Henderson GR, Vander Meer RK, Breed MD, Espelie KE, Winston ML (1998). Primer pheromones and possible soldier caste influences on the evolution of sociality in lower termites.. *Pheromone Communication in Social Insects*.

[bibr07] Horiuchi S, Yamamura N, Abe T (2002). Soldier production strategy in lower termites: from young instars or old instars?. *Journal of Theoretical Biology*.

[bibr08] Howard RW, Haverty MI (1981). Production of soldiers and maintenance of soldier proportion by laboratory experimental groups of *Reticulitermes flavipes* (Kollar) and *Reticulitermes virginicus* (Banks) (Isoptera: Rhinotermitidae).. *Insect Sociaux*.

[bibr09] Lenz M, Luscher M (1976). The dependence of hormone effects in caste determination on external factors.. *Phase and Caste Determination in Insects: Endocrine Aspects.*.

[bibr10] Liu Y, Henderson G, Mao L, Laine RA (2005). Seasonal variation of juvenile hormone titers of the formosan subterranean termite, *Coptotermes formosanus* (Rhinotermitidae).. *Environmental Entomology*.

[bibr11] Mao L, Henderson G, Lui Y, Laine RA (2005). Formosan subterranean termite (Isoptera: Rhinotermitidae) soldiers regulate juvenile hormone levels and caste differentiation in workers.. *Annals of the Entomological Society of America*.

[bibr12] Park YI, Raina AK (2004). Juvenile hormone III titers and regulation of soldier caste in *Coptotermes formosanus*.. *Journal of Insect Physiology*.

[bibr13] Park YI, Raina AK (2005). Regulation of juvenile hormone titers by soldiers in the Formosan subterranean termite, *Coptotermes formosanus*.. *Journal of Insect Physiology*.

[bibr14] Raina AK, Park YI, Gelman D (2008). Molting in workers of the Formosan subterranean termite *Coptotermes formosanus*.. *Journal of Insect Physiology*.

[bibr15] Scharf ME, Buckspan CE, Grzymala TF, Zhou X (2007). Regulation of polyphenic differentiation in the termite *Reticulitermes flavipes* by interaction of intrinsic and extrinsic factors.. *Journal of Experimental Biology*.

[bibr16] Scharf ME, Ratliff CR, Hoteling JT, Pittendrigh BR, Bennett GW (2003). Caste differentiation responses of two sympatric *Reticulitermes* termite species to juvenile hormone homologs and synthetic juvenoids in two laboratory assays.. *Insectes Sociaux*.

[bibr17] Scharf ME, Wu-Scharf D, Pittendrigh BR, Bennett GW (2003). Caste— and development— associated gene expression in a lower termite.. *Genome Biology*.

[bibr18] Su N-Y (2002). Novel Technologies for Subterranean Termite Control.. *Sociobiology*.

[bibr19] Szalanski AL, Austin JW, Owens CB (2003). Identification of *Reticulitermes* spp. (Isoptera: Reticulitermatidae) from South Central United States by PCR-RFLP.. *Journal of Economic Entomology*.

[bibr20] Tarver MR, Schmelz EA, Rocca JR, Scharf ME (2009). Effects of soldier—derived terpenes on soldier caste differentiation in the termite *Reticulitermes flavipes*.. *Journal of Chemical Ecology*.

[bibr21] Tarver MR, Zhou X, Scharf ME (2010). Socio— environmental and endocrine influences on developmental and caste—regulatory gene expression in the eusocial termite *Reticulitermes flavipes*.. *BMC Molecular Biology*.

[bibr22] Waller DA, La Fage JP (1988). Environmental influence on soldier differentiation in *Coptotermes formosanus*.. *Insectes Sociaux*.

[bibr23] Waibel M, Floreano D, Magnenat S, Keller L (2006). Division of labour and colony efficiency in social insects: effects of interaction between genetic architechture, colony kin structure and rate of perturbations.. *Proceedings of the Royal Society of London B–Biological Sciences*.

[bibr24] Wilson EO (1971). *The Insect Societies.*.

